# Neural basis of affective touch and pain: A novel model suggests possible targets for pain amelioration

**DOI:** 10.1111/jnp.12250

**Published:** 2021-05-12

**Authors:** Larissa L. Meijer, Carla Ruis, Maarten J. van der Smagt, Erik J. A. Scherder, H. Chris Dijkerman

**Affiliations:** ^1^ Utrecht University The Netherlands; ^2^ University Medical Centre Utrecht The Netherlands; ^3^ Vrije Universiteit Amsterdam The Netherlands

**Keywords:** pain, affective touch, CT‐afferents, chronic pain

## Abstract

Pain is one of the most common health problems and has a severe impact on quality of life. Yet, a suitable and efficient treatment is still not available for all patient populations suffering from pain. Interestingly, recent research shows that low threshold mechanosensory C‐tactile (CT) fibres have a modulatory influence on pain. CT‐fibres are activated by slow gentle stroking of the hairy skin, providing a pleasant sensation. Consequently, slow gentle stroking is known as affective touch. Currently, a clear overview of the way affective touch modulates pain, at a neural level, is missing. This review aims to present such an overview. To explain the interaction between affective touch and pain, first the neural basis of the affective touch system and the neural processing of pain will be described. To clarify these systems, a schematic illustration will be provided in every section. Hereafter, a novel model of interactions between affective touch and pain systems will be introduced. Finally, since affective touch might be suitable as a new treatment for chronic pain, possible clinical implications will be discussed.

## Background

Pain is a fascinating phenomenon; it can be the friend that protects us from harm, but it can also be the enemy that makes us suffer. For this reason, pain has been studied extensively over the last century. We now have substantial knowledge about the neural processing of pain (Bourne, Machado, & Nagel, 2014). Unfortunately, many people still suffer from (chronic) pain. In the United States, approximately 19–43% of the adult population suffers from chronic pain (classified as, when pain lasts longer than 3 months; Pitcher, Von Korff, Bushnell, & Porter, 2019), in the United Kingdom 33–50% (Fayaz, Croft, Langford, Donaldson, & Jones, 2016) and in Latin‐American, Asian and African countries the incidence of chronic pain is estimated between 13 and 51% (Sá et al., 2019). These statistics underline the fact that chronic pain is a major health problem. Chronic pain severely impacts mental health, leading to conditions such as depression, anxiety, anhedonia and impacts quality of life in general (Hylands‐White, Duarte, & Raphael, 2017; Simons, Elman, & Borsook, 2014). In addition, the prevalence of painful conditions, for example osteoarthritis and lower back pain, might increase with ageing and since the general population is getting older, more people will suffer from chronic pain in the near future (Schwan, Sclafani, & Tawfik, 2019). All these factors highlight the importance of finding new ways to reduce pain.

Interestingly, recent research suggests that affective touch might be a possible candidate for pain amelioration. Affective touch is gentle stroking of the skin which provides a pleasant sensation (Björnsdotter, Morrison, & Olausson, 2010). This type of touch activates a particular type of low threshold mechanosensory C‐fibres (C‐tactile or CT‐afferents), which appear to modulate pain (Liljencrantz et al., 2017). CT‐afferents can be activated by slow stroking with a soft brush or with the hand, between 1 and 10 cm/s (optimal activation at 3 cm/s), and is therefore also referred to as CT‐optimal touch (Björnsdotter et al., 2010). Recent behavioural and neurophysiological research confirms that the CT‐afferent system and pain are connected. CT‐optimal touch appears to be effective in reducing acute pain (Gursul et al., 2018; Habig et al., 2017; Liljencrantz et al., 2017; von Mohr, Krahé, Beck, & Fotopoulou, 2018). This finding makes CT‐optimal touch a promising candidate for a new pain intervention, which could be especially helpful for people suffering from chronic pain conditions as adequate treatments are lacking.

A clear overview of the neural mechanisms that could be involved in the modulatory effects of CT‐optimal touch on pain is missing in the present literature. This review aims to resolve this gap by describing the neural basis of the CT‐afferent system, an overview of the pain system, and the neural interaction between these two somatosensory modalities. As CT‐optimal touch might be a promising candidate to reduce chronic pain, we will subsequently discuss the possible interaction between CT‐optimal touch and chronic pain. In addition, clinical implications for chronic pain reduction will be discussed.

## The neurophysiology of affective touch and pain

### Affective touch/CT‐optimal touch

The skin is our largest organ and helps us to engage with the world. It is innervated by three types of sensory nerve fibres, A‐Beta (Aβ), A‐Delta (Aδ) and C‐fibres, which mediate our somatosensations (Zimmerman, Bai, & Ginty, 2014). Historically, tactile sensibility (touch) was thought to be signalled exclusively through fast conducting (50 m/s) myelinated Aβ‐fibres. Aβ‐fibres have a high spatial and temporal resolution and are linked to the discriminative aspects of touch (McGlone, Wessberg, & Olausson, 2014).

In contrast, affective touch concerns the more affective and pleasant aspects of touch and activates a subgroup of C‐fibres known as C‐tactile (CT) fibres (Björnsdotter et al., 2010). CT‐fibres are unmyelinated slow conducting afferents and have a low temporal and spatial resolution (Vallbo, Olausson, & Wessberg, 1999). The CT‐afferents respond to innocuous stimuli such as slow stroking of the hairy skin (most effectively between 1 and 10 cm/s, optimal speed is 3 cm/s), which can be applied with a soft brush or hand (Ackerley, Carlsson, Wester, Olausson, & Backlund Wasling, 2014; Olausson, Wessberg, Morrison, McGlone, & Vallbo, 2010). Moreover, the CT‐system responds most vigorously to tactile stimuli that are around 34°C, that is skin temperature (Ackerley et al., 2018). As an optimal stroking speed of 3 cm/s is required to activate the CT‐fibres, this type of touch is also referred to as CT‐optimal touch. Since this review simply focuses on the underlying mechanisms of affective touch, rather than the perceived pleasantness and social component, the term CT‐optimal touch will be used from hereon.

**Figure 1:**
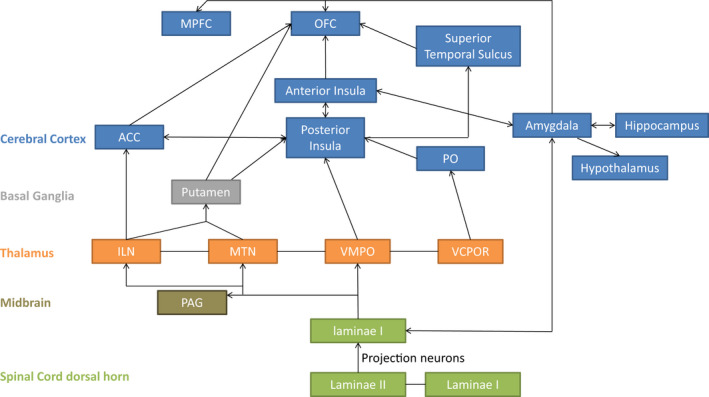
Schematic overview of neuronal projections of the CT‐afferent system. PAG = periaqueductal Grey; ILN = intralaminar thalamic nuclei; MTN = medial thalamic nuclei; VMPO = ventral medial posterior thalamic nuclei; VCPOR = ventral caudal portae thalamic nuclei; PO = parietal operculum; ACC = anterior cingulate cortex; OFC = orbitofrontal cortex; MPFC = medial prefrontal cortex. This figure is based on the following literature: Beauchamp et al. (2008), Craig (2002, 2009), Craig, Chen, Bandy, and Reiman (2000), Gordon et al. (2013), Marshall and McGlone (2020), Morrison (2016), Olausson et al. (2008), Sailer et al. (2016).

Recent research has focused on the underlying neural pathway of the CT‐fibres. As this has already been described thoroughly in a state of the art review of McGlone et al. (2014), only a short overview and more recent insights will be provided here. A schematic overview of the CT‐system is shown in Figure 1.

The CT‐fibres transmit signals to the superficial laminae I and II of the spinal cord dorsal horn; from thereon the signal is conveyed to several medial and intralaminar thalamic nuclei. It is thought that transmission occurs through the spinothalamic tract (STT; McGlone et al., 2014). However, recent research shows that spinothalamic ablation does not affect the CT‐system, suggesting that the CT‐afferents possibly project through the dorsal column of the spinal cord to the thalamic nuclei (Marshall, Sharma, Marley, Olausson, & McGlone, 2019). Furthermore, animal research suggests that CT‐afferents access the dorsal column through an interneuronal zone between laminae II and V (Abraira et al., 2017). However, it is currently not completely clear how the CT‐afferents are projected to the thalamus, but multiple ascending pathways may be involved (Marshall & McGlone, 2020). At a cortical level, several regions are activated, starting with the posterior insula and, from there, the anterior insula, anterior cingulate cortex (ACC), superior temporal sulcus, orbitofrontal cortex (OFC), medial prefrontal cortex (MPFC), amygdala, hippocampus and hypothalamus are activated (Figure 1; Beauchamp, Yasar, Frye, & Ro, 2008; Craig, 2002, 2009; Gordon et al., 2013; Morrison, 2016; Sailer et al., 2016). As mentioned, the CT‐system is linked to the affective experience of touch. The activation of especially the insula and ACC account for this affective component (Gordon et al., 2013). In addition, the OFC and MPFC are linked to our (social) reward system, which supports their function in the affective (rewarding) aspects of this type of touch (Gordon et al., 2013; von Mohr, Crowley, et al., 2018).

### Pain

Pain is defined as ‘a complex sensory and emotional experience associated with actual or potential tissue damage, or described in terms of such damage’ (International Association for the Study of Pain; Bell, 2018). Pain can be divided into acute and chronic pain. Acute pain is regarded as a normal reaction to a harmful stimulus. Acute pain warns us that something is wrong and therefore plays a necessary and protective role. When pain exceeds its normally stated healing time and is present for at least 3 months, it is classified as chronic pain (Świeboda, Filip, Prystupa, & Drozd, 2013). Chronic pain is seen as a disease on its own and has a severe impact on quality of life, affecting physical and mental functioning (Anwar, 2016).

**Figure 2:**
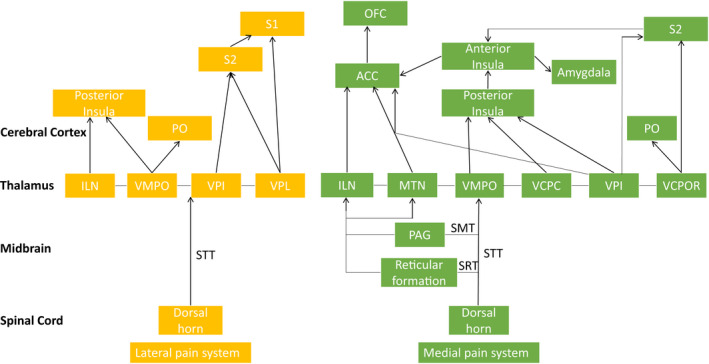
Schematic overview of (sub)cortical areas activated by the lateral and medial pain system. The lateral pain system is illustrated in yellow and the medial pain system in green. STT = spinothalamic tract; SRT = spinoreticular tract; SMT = spinomesencephalic tract; ILN = intralaminar nuclei; MTN; medial thalamic nuclei; VMPO = ventral medial posterior thalamic nuclei; VCPC; ventral caudal parvocellular nucleus; VPI; ventro posterior inferior nucleus; VCPOR = ventral caudal portae thalamic nuclei; VPL = ventral posterolateral nucleus; PO = parietal operculum; ACC = anterior cingulate cortex; OFC = orbitofrontal cortex; S2 = secondary somatosensory cortex; S1 = primary somatosensory cortex. This figure is based on the following literature: Apkarian et al. (2005), Bourne et al. (2014), Craig, Bushnell, Zhang, and Blomqvist (1994), Fenton, Shih, and Zolton (2015), Garcia‐Larrea and Peyron (2013), Lenz et al. (2004), Lu et al. (2016), Peirs and Seal (2016), Peyron et al. (2000), Scherder et al. (2003), Schweinhardt and Bushnell (2010), Sewards and Sewards (2002), Vogt and Sikes (2000), Woller et al. (2017).

The neural mechanisms underlying acute pain have already been described in reviews by Bell (2018) and Hudspith (2016). Therefore, only a short overview and schematic illustration will be presented. Painful or noxious stimuli are transmitted by Aβ‐, Aδ‐ and C‐fibres to the dorsal horn of the spinal cord. From here, a distinction between the lateral‐ and medial pain system can be made, illustrated in Figure 2. The Aβ‐ and Aδ‐fibres project through the STT to the ventral thalamic nuclei and are part of the lateral pain system. These thalamic nuclei project directly to the secondary somatosensory cortex (S2), the primary somatosensory cortex (S1), insula and parietal operculum (PO; Apkarian, Bushnell, Treede, & Zubieta, 2005; Lenz, Weiss, Ohara, Lawson, & Greenspan, 2004; Peyron, Laurent, & Garcia‐Larrea, 2000; Scherder, Sergeant, & Swaab, 2003). This system carries information about the sensory‐discriminative aspects of pain (Woller, Eddinger, Corr, & Yaksh, 2017).

The C‐fibres, on the other hand, project through the STT to the medial and intralaminar thalamic nuclei and are part of the medial pain system. This system carries information about the motivational‐affective aspects of pain (Scherder et al., 2003; Sewards & Sewards, 2002; Vogt & Sikes, 2000). Through the thalamic nuclei the posterior insula, anterior insula, ACC, S2, PO, amygdala and OFC are innervated (Garcia‐Larrea & Peyron, 2013; Lu et al., 2016; Peyron et al., 2000; Schweinhardt & Bushnell, 2010). Especially, the anterior insula and the ACC appear necessary for the affective components of pain (Apkarian et al., 2005; Lu et al., 2016; Peyron et al., 2000; Sewards & Sewards, 2002; Vogt & Sikes, 2000).

## Interaction between CT‐optimal touch and acute pain

Pain and touch are closely related sensory modalities. Behaviourally, this is evident by the way we react to a painful stimulus. For instance, when we stub our toe, we tend to rub or stroke the part that hurts, to reduce the painful sensation. This reaction can be explained by the gate control theory, which is based on the notion that at the spinal level there is a ‘gate’ which can be ‘closed’ by activation of large diameter fibres (Aβ‐fibres), for example rubbing, and thereby preventing the pain stimulus of reaching the cortex (Melzack & Wall, 1965). However, this theory is criticized, as its representation of the neural architecture of the spinal cord and the modulatory system exhibits oversimplifications and flaws (Moayedi & Davis, 2013). For example, the modulatory system of the Gate Control Theory does not include descending small fibres from the brainstem, which, as we now know, do play an important role in pain modulation (Moayedi & Davis, 2013). Interestingly, there are also other types of touch associated with pain relief, namely: massage, handholding and affective touch (i.e., CT‐optimal touch; Reddan, Young, Falkner, López‐Solà, & Wager, 2020). Their common factors are the affective and pleasant sensation that they elicit and the strong social component, hence they have also been described as interpersonal‐ or social touch (Goldstein, Weissman‐Fogel, Dumas, & Shamay‐Tsoory, 2018). Recent research shows that interpersonal touch influences our well‐being and can reduce stress and acute pain (López‐Solà, Geuter, Koban, Coan, & Wager, 2019). Furthermore, interpersonal touch provides a feeling of social support which is also associated with a reduction of pain intensity in chronic pain and cancer patients (Goldstein et al., 2018).

Massage is possibly the most common form of interpersonal touch and often used to reduce soreness of muscles and back pain. Studies into pain modulation through massage therapy mostly focused on reducing back pain in adults (Field, 2019). Multiple mechanisms underlying pain modulation through massage have been described, the most common of which is the aforementioned Gate Control Theory wherein deep pressure massage activates the fast conducting Aβ‐fibres (Field, Diego, & Hernandez‐Reif, 2007). In addition, deep pressure massage is associated with an increase in vagal activity which reduces levels of cortisol which, in turn, leads to a reduction in pain (Field, 2014).

Another form of interpersonal touch is handholding. Current literature does not describe the underlying peripheral mechanism of handholding, but since it mostly involves touch on the glaborous skin, Aβ‐fibres are probably involved. Recent research into handholding shows that handholding a partner can indeed reduce pain (Goldstein et al., 2018; Goldstein, Weissman‐Fogel, & Shamay‐Tsoory, 2017; López‐Solà et al., 2019; Reddan et al., 2020). In addition, the study of Goldstein et al. (2017) shows that during handholding, pain receiver and hand holder both show respiration and heart rate coupling, that is interpersonal physiological coupling, resulting in shared empathy for pain and emotional support. Furthermore, fMRI and EEG data showed that brain‐to‐brain coupling also occurs during handholding (Goldstein et al., 2018). Brain areas associated with reward, affection and emotional state are activated in both giver and receiver (Goldstein et al., 2018). The feeling of social and emotional support through handholding is associated with activation of the reward circuitry which has been linked to pain reduction. For instance, brain regions involved in the rewarding circuitry, for example OFC and dorsolateral prefrontal cortex (PFC) have been shown to project to descending pain modulatory systems (Younger, Aron, Parke, Chatterjee, & Mackey, 2010). Thus, the analgesic effect of handholding may be explained by social understanding and support, which is rewarding and results in pain reduction (Goldstein et al., 2018; López‐Solà et al., 2019; Reddan et al., 2020).

The third and more recently discovered form of interpersonal touch is CT‐optimal touch, that is a gentle stroking of the skin at 3 cm/s (McGlone et al., 2014). Recent behavioural research shows that CT‐optimal touch modulates acute pain experience. Habig et al. (2017) focused on the effect of CT‐optimal touch on pain in healthy individuals compared to small fibre neuropathy (SFN) patients. SFN targets the thinly myelinated nerve fibres (C‐fibres) and it is therefore hypothesized that the CT‐fibres are impaired in this group. All participants underwent three conditions: heat pain only, CT‐optimal touch only and heat pain combined with CT‐optimal touch. Results show that CT‐optimal touch reduces pain in healthy individuals, while the SFN patients do not experience a reduction in pain. Since the CT‐fibres are not intact in these patients, this further confirms that CT‐optimal touch can modulate pain through activation of the CT‐fibres. However, an important limitation of the study by Habig et al. (2017) is the lack of a control touch condition.

Another study into the effect of CT‐optimal touch on acute pain did use touch as a control condition and therefore provides more support for the CT‐fibres’ pain modulating role. Liljencrantz et al. (2017) also used a heat pain stimulus to induce pain in healthy participants, while they simultaneously received CT‐optimal touch, CT non‐optimal touch (i.e., fast stroking of the skin) or vibration on the skin. Results show that CT‐optimal touch significantly reduces acute pain experience compared to fast non‐optimal CT‐stroking or vibration on the skin (Liljencrantz et al., 2017). The results of this study are consistent with a role of the CT‐fibre system in pain modulation and suggest a less important role for the Aβ‐fibres in pain modulation through touch. In an additional experiment conducted by Liljencrantz et al. (2017), participants received a heat pain stimulus and CT‐optimal touch, but temporal spacing between the two types of stimulation varied. The results show that pain relief was most pronounced when CT‐optimal touch was applied directly before the heat pain stimulus compared to longer intervals. Furthermore, peak pain ratings are significantly lower during long stroking duration compared to short stroking duration. This suggests that the analgesic effect of CT‐optimal touch does not depend on any possible distraction from the pain stimulus, when touch is applied (Liljencrantz et al., 2017).

In addition to these studies in adults, Gursul et al. (2018) investigated the effects of CT‐optimal touch on pain experience in infants, who received a clinical heel lance for blood collection. Ten seconds prior to the heel lance one group received CT‐optimal touch and one group received no touch. To measure behavioural responses, the pain‐related facial expression was recorded. Results show that both groups exhibited facial grimacing, but the duration was 50% shorter for infants receiving CT‐optimal touch. Compared to research in which pain was experimentally induced, this research shows that CT‐optimal touch can also reduce experienced pain during a medical procedure. In sum, these behavioural studies indicate that CT‐optimal touch can reduce acute pain experience in adults and infants.

**Figure 3:**
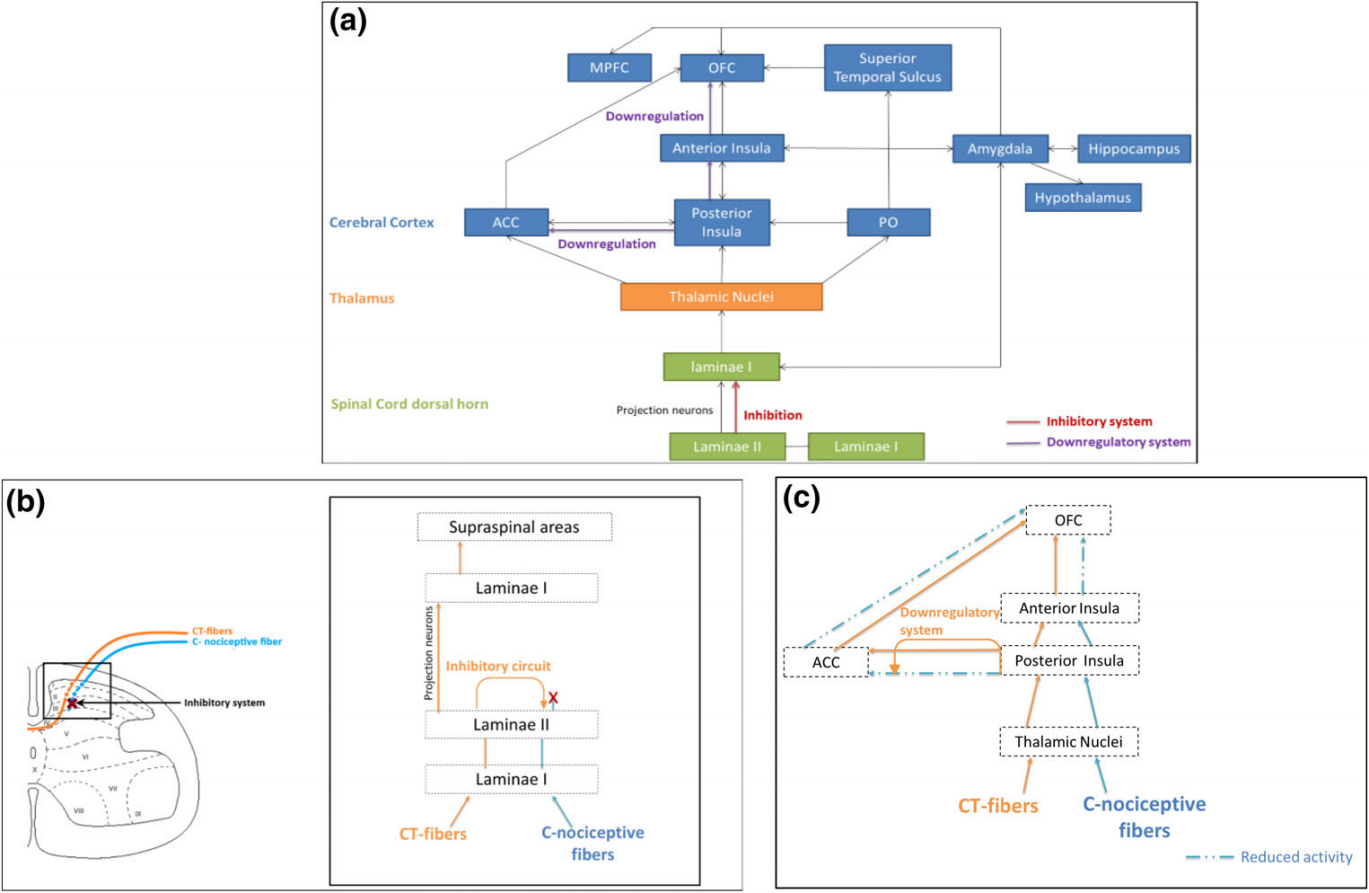
(a) schematic overview of the CT‐afferent system including the two ways of pain modulation. The red line represents the inhibitory system within the dorsal horn. The purple line represents the downregulating system within cortical brain areas. (b) Dorsal horn inhibitory system. C. Cortical downregulatory system. ACC = anterior cingulate cortex; PO = parietal operculum; OFC = orbitofrontal cortex; MPFC = medial prefrontal cortex. Figure is based on the following literature: Gursul et al. (2018), Habig et al. (2017), Liljencrantz et al. (2017), Lu and Perl (2003), Marshall and McGlone (2020), Shaikh et al. (2015), von Mohr, Krahé, et al. (2018).

In an effort to understand the neurophysiology behind these behavioural effects of CT‐optimal touch on pain, several studies suggest that the CT‐afferent system can modulate pain through a bottom‐up process starting in the dorsal horn of the spinal cord (Gursul et al., 2018; Habig et al., 2017; Krahé, Drabek, Paloyelis, & Fotopoulou, 2016; Lu & Perl, 2003; von Mohr, Krahé, et al., 2018). Furthermore, the CT‐afferent system activates several brain areas, for example the insula and ACC, that are not only associated with the affective and subjective evaluation of touch, but also with the subjective appreciation of pain, that is the medial pain system (illustrated in Figures 1 and 2). Therefore, it could be that pain modulation by the CT‐system also occurs at supraspinal levels. This implies that there are possibly two ways through which the CT‐afferents can modulate pain processing, referred to as the inhibitory system and the downregulating system. This is illustrated in Figure 3.

First, electrophysiological research in animals shows that neurons within laminae II of the spinal dorsal horn contain a specific inhibitory pathway related to CT‐afferent input (Lu & Perl, 2003). The laminae II neurons activated by CT‐afferent projections inhibit laminae II neurons receiving nociceptive input. This prevents nociceptive input from reaching laminae I and thereby (sub)cortical brain regions involved in pain processing. This inhibitory circuit could represent the way innocuous impulses suppress nociceptive impulses (Habig et al., 2017; Liljencrantz et al., 2017; Lu & Perl, 2003).

Second, several human studies show that the CT‐afferent system also modulates the activation of brain areas related to pain processing. In addition to the behavioural experiment of Gursul et al. (2018), they investigated the effect of CT‐optimal touch versus CT non‐optimal touch on noxious‐evoked brain activity measured with EEG in infants who received a pinprick. Results show that CT‐optimal touch significantly reduces the magnitude of noxious evoked brain activity compared to CT non‐optimal touch. Furthermore, Krahé et al. (2016) studied the effect of CT‐optimal touch versus CT non‐optimal touch on laser‐evoked potentials (LEP’s) to noxious stimulation. The results show that CT‐optimal touch reduces the LEP’s local peak amplitude on the N1 complex. The N1 reflects early stages of pain processing mostly occurring outside conscious awareness. They find no effect of CT‐optimal touch on the N2‐P2 complex, which is thought to reflect higher order processing of pain, mostly associated with the socio‐cognitive aspects of pain experience (Krahé et al., 2015, 2016). Based on the study of Krahé et al. (2016), von Mohr, Krahé, et al. (2018) investigated the effect of CT‐optimal touch versus CT non‐optimal touch applied *by a romantic partner* on laser‐evoked potentials (LEP’s) to noxious stimulation. CT‐optimal touch significantly reduces the LEP’s local peak amplitude on the N1 as well as the N2‐P2 complex. As mentioned, the N2‐P2 complex is associated with higher order conscious processing of pain, mostly linked to activity in the anterior insula and the ACC, considered important for the motivational and affective aspects of pain (von Mohr, Krahé, et al., 2018). The reduced LEP’s peak in the N2‐P2 complex suggests that when CT‐optimal touch is applied by a romantic partner together with a noxious stimulus, the pain related processing in the anterior insula and ACC show downregulation, which may modulate the motivational aspects of pain (Habig et al., 2017; Shaikh, Nagi, McGlone, & Mahns, 2015; von Mohr, Krahé, et al., 2018).

However, fMRI data from the study of Habig et al. (2017) appears inconsistent with the findings of von Mohr, Krahé, et al. (2018) and Krahé et al. (2016). Here, no significant differences in cortical activation were found between noxious stimulation with and without CT‐optimal touch, even though participants did report a reduction in pain when CT‐optimal touch was applied (Habig et al., 2017). Therefore, it may be argued that the downregulation of the N1 and N2‐P2 complexes, as demonstrated by von Mohr, Krahé, et al. (2018) reflects pain modulation through the aforementioned bottom‐up processes in the spinal dorsal horn. However, in the same study of von Mohr, Krahé, et al. (2018) pain modulation through CT‐optimal touch could not be based on the inhibitory circuitry within the spinal dorsal horn. In this study, the tactile stimulus and pain stimulus were delivered at different times and different body parts, which were therefore unlikely to interact at spinal levels, providing evidence for a pain modulating role of the CT‐system through higher order mechanisms in the insula and ACC. In addition, this study also showed that the effectiveness of pain modulation through CT‐fibre stimulation depends on social factors and perceived feelings of social support. This is in line with previous research suggesting that the perceived pleasantness of CT‐optimal touch is linked to the affective and interpersonal properties of this kind of touch (McGlone et al., 2014). Moreover, research into pleasure‐related analgesia reveals that pleasurable sensations provide top‐down modulation of nociception (Leknes & Tracey, 2008), which may be linked to PFC and insula activation, regions also strongly involved in CT‐optimal touch (Leknes & Tracey, 2008; Morrison, 2016). Given the strong connection between CT‐fibre activation and perceived pleasantness of the touch (Björnsdotter et al., 2010), it could be that the CT‐system also reduces pain through top‐down pleasure‐related analgesia.

Taken together, these studies provide substantial behavioural and neural evidence supporting a pain modulating role for CT‐optimal touch. Based on these studies, a novel model illustrating the neurophysiology of the CT‐afferent system, and its pain amelioration can be introduced (see Figure 3). Figure 3b shows the proposed inhibitory system within the dorsal horn of the spinal cord. This system inhibits the pain stimulus from reaching ascending pathways and thereby prevents further cortical processing, resulting in pain reduction. Furthermore, Figure 3c illustrates pain modulation through downregulation of the insula and ACC, both important for the processing of the subjective experience of pain. Currently, it is unclear whether this downregulation is a result of the bottom‐up inhibitory system – that is the inhibitory system prevents the pain stimulus from reaching the brain resulting in reduced activation at cortical levels measured with EEG – or the result of modulation through the insula and ACC itself. Further research into the exact neural mechanism should clarify the contradictory evidence for modulation at a cortical level.

As described previously, CT‐optimal touch is not the only type of interpersonal touch associated with pain reduction. However, compared to CT‐optimal touch, the pain modulating role of massage therapy seems to be based on different processes. Unfortunately, there are no studies into the neurophysiology of massage, and the studies that have been conducted suffer from several methodological limitations which makes it difficult to understand the underlying mechanism of massage (Field, 2019).

In contrast, handholding and CT‐optimal touch appear to rely on similar cortical processes for pain modulation. Both types of touch are interpersonal‐social types of touch and depend on the activation of brain areas associated with affection and reward, which are important for their pain modulating role (Krahé et al., 2016; López‐Solà et al., 2019). Hypothetically, it is possible that these two types of interpersonal touch rely partially on the same social and affective brain network. CT‐optimal touch relies on direct CT‐fibre input, thereby activating this affective network. Handholding may rely on indirect activation of this affective network through the social and affective aspects of this kind of touch. Interestingly, recently published research shows that CT‐afferents not only innervate the human hairy skin but also the glaborous skin of the hand (Watkins et al., 2020). Although the density is much lower than in hairy skin, it could explain why slowly touching the palm of the hand is also perceived as pleasant and why handholding reduces pain (Watkins et al., 2020).

### Clinical implications

The described modulating role of the CT‐system on acute pain experience raises the question: might CT‐optimal touch also reduce chronic pain?

The underlying mechanisms of chronic pain are still not completely understood, but studies do show that in musculoskeletal pain, osteoarthritis and neuropathic pain there are changes in the structural and functional connectivity of brain regions involved in pain processing. Especially the insula, ACC and PFC appear to show changes in connectivity which are linked to an increase in pain intensity and clinical pain duration (Kuner & Flor, 2016; Schmidt‐Wilcke, 2015). Because of the mostly unknown underlying mechanisms of chronic pain, it is hard to find a suitable treatment. Currently, treating chronic pain is based on a multimodal approach in which pharmacological, non‐pharmacological and physical rehabilitation are combined. Unfortunately, there are still many people suffering from chronic pain (Bicket & Mao, 2015).

Based on the presented research, CT‐optimal touch could be a promising candidate in reducing chronic pain. Indeed, a recently published paper of Di Lernia, Lacerenza, Ainley, and Riva (2020) shows that CT‐optimal touch significantly reduces the severity of reported pain in chronic pain patients by 23% after 11 min of stimulation. Participants suffered from primary chronic pain, secondary musculoskeletal pain and neuropathic pain and received either CT‐optimal touch or vibration on the skin. The effect of CT‐optimal touch was independent of pathological condition (Di Lernia et al., 2020). Even in central and peripheral neuropathic pain its severity appears reduced by CT‐optimal touch. This is unexpected since research also links CT‐fibre stimulation to tactile allodynia, a symptom of neuropathic pain in which innocuous stimuli elicit a painful burning sensation. Since CT‐optimal touch is gentle stroking of the skin this could elicit tactile allodynia (Nagi, Rubin, Chelvanayagam, Macefield, & Mahns, 2011). However, even before CT‐fibres were discovered, it was suggested that Aβ‐fibres elicit allodynia following central sensitization in the dorsal horn, a notion that is also suggested by recent research (Liljencrantz & Olausson, 2014). This could explain why CT‐optimal touch and skin vibration did not elicit a painful sensation in the study of Di Lernia et al. (2020) and, more importantly, why CT‐optimal touch reduced the experienced chronic pain. As described in the previous section and illustrated in Figure 3, pain modulation through the CT‐system may depend on multiple neural mechanisms that may downregulate the possible overactivation of the ACC and PFC in chronic pain resulting in a decrease in experienced pain severity (Gursul et al., 2018; Krahé et al., 2016; Lu & Perl, 2003; Schmidt‐Wilcke, 2015; von Mohr, Crowley, et al., 2018). Overall, CT‐optimal touch seems very promising for reducing chronic pain.

Therefore, it would be of interest to study whether CT‐optimal touch can reduce chronic pain in other clinical patient groups. In neurodegenerative diseases, chronic pain is very common. In mild to moderate stages of Alzheimer’s Disease (AD), 38–75% are suffering from chronic pain. It seems that the descending pain pathways are affected leading to an increase in pain (de Tommaso et al., 2016). Given the course of AD, it is expected that the CT‐fibres are intact as these systems are unaffected, however, this has not been studied yet. So, in AD CT‐optimal touch could alleviate pain, but only in mild to moderate stages as in later stages ascending pathways seem affected leading to a reduction in pain (de Tommaso et al., 2016). Another patient group suffering considerably from chronic pain is Multiple Sclerosis (MS), with a prevalence of 50–86% (de Tommaso et al., 2016). The underlying mechanisms causing pain in MS are not yet understood, but it seems plausible that there are alterations in the pain network caused by demyelization (Borsook, 2012). We argue that the CT‐fibres are still intact in MS, as they are not myelinated. If CT‐optimal touch could modulate pain in MS it is more likely to occur at the dorsal horn of the spinal cord, because demyelization could also affect cortical areas related to CT‐optimal touch. Finally, in Parkinson’s Disease (PD) 30–95% are suffering from chronic pain (Blanchet & Brefel‐Courbon, 2018). This is caused by overactivation of regions involved in pain processing, especially the ACC and insula (Antonini et al., 2018; Tseng & Lin, 2017). Interestingly, a recent study revealed that PD patients, similar to healthy participants, report higher pleasantness ratings for CT‐optimal stroking velocities compared to higher or lower stroking velocities (Kass‐Iliyya et al., 2017). This suggests that CT‐optimal touch is perceived and processed in the same way in PD patients as in healthy controls. This finding makes CT‐optimal touch a promising candidate to reduce pain in PD.

Overall, based on the aforementioned studies, CT‐optimal touch may reduce pain in these patient groups and may therefore be useful as a new, alternative or supplementary pain intervention (Di Lernia et al., 2020; Gursul et al., 2018; Habig et al., 2017; Liljencrantz et al., 2017; von Mohr, Krahé, et al., 2018). If proven effective, it may be implemented in daily care routines in which a partner or caregiver provides CT‐optimal touch, as this appears to increase its beneficial effects (von Mohr, Krahé, et al., 2018). Based on the duration of perceived pleasantness of CT‐optimal touch, a duration of approximately 10–15 min is proposed (Sailer et al., 2016). This kind of intervention can take place from home and it does not involve trained therapist, which makes it easy to apply and implement in daily life. Based on the aforementioned studies, CT‐optimal touch may not diminish pain completely, it is therefore more likely that it can be used complementary to existing pain treatments.

### Conclusion

In summary, pain and CT‐optimal touch (affective touch) depend on partially overlapping neural mechanisms. Recent research has focused on the neural process underlying CT‐optimal touch and how they possibly influence the processing of pain and pain experience. Several studies show that CT‐optimal touch can reduce acute pain experience, and a few studies have investigated the underlying neurophysiological mechanism for this modulating role of CT‐optimal touch. With the current review, we aimed to provide an overview of recent research and knowledge about affective touch and pain, and how they can interact. The latter is illustrated by a novel model (Figure 3).

This modulating function of CT‐optimal touch makes it a promising candidate for new interventions. Importantly, recent experimental research shows that CT‐optimal touch can reduce chronic pain in a variety of patient groups. Based on these findings, it would be interesting to study whether CT‐optimal touch could also be implemented as a treatment for chronic pain as there are several clinical populations, for whom current pain treatments are not sufficient.

### Search strategy and selection criteria

**Figure 4:**
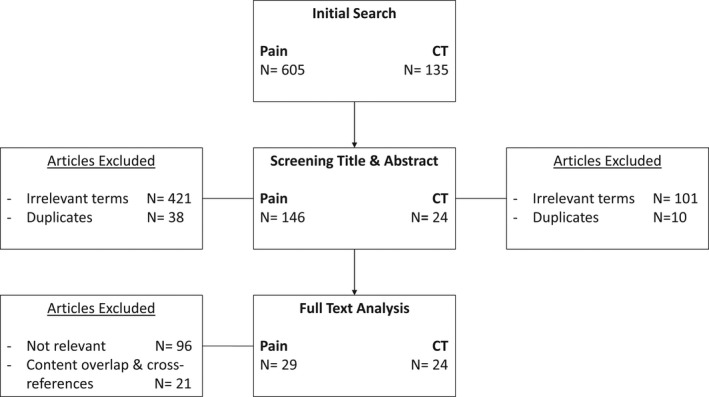
Flowchart illustrating the screening and selection process for paper inclusion.

A literature search was conducted to find relevant articles on pain and affective/CT‐optimal touch (Figure 4). The following databases were used: PubMed, Embase and Chochrane. For the pain literature as selection criteria, the following search terms were used: pain physiology, pain perception, nociceptor physiology, nociception and chronic pain. Including filters: publication last 10 years, review, human species. This provided 605 articles. Subsequently, the title and abstract were screened based on the following selection criteria: acute pain, chronic pain, physiology, pathophysiology and anatomy. In addition, literature focusing on specific diseases and/or pain syndromes (e.g., migraine, musculoskeletal pain) were excluded. This led to exclusion of 421 articles. There were 38 articles excluded as these were duplicates. This led to *N* = 605 − 459 = 146 possible relevant articles. The full text of these 146 articles was analysed to determine relevancy, resulting in 50 articles selected. Because of content overlap within certain articles and cross references, 29 were eventually used.

For the affective/CT‐optimal touch literature, the following search terms were used: affective touch, gentle touch, CT‐afferents. No filters were added. This provided 135 articles. For title and abstract screening, the following inclusion criteria were applied: physiology, brain, cortical, processing; as well as the following exclusion criteria: social touch, infants. This resulted in 34 articles (i.e., 101 articles were excluded). Ten articles were duplicates and excluded as well. Based on abstract and/or full text analyses, all 24 remaining articles were relevant and used for this review.

A literature search on ‘affective touch and pain’ and ‘CT fibres and pain’ resulted in eight additional relevant articles.

## Conflicts of interest

All authors declare no conflict of interest.

## Author contribution

Larissa Lauren Meijer, MSc. (Conceptualization; Funding acquisition; Writing – original draft; Writing – review & editing) C. Ruis (Conceptualization; Supervision; Writing – original draft; Writing – review & editing) M. J. van der Smagt (Conceptualization; Funding acquisition; Supervision; Writing – original draft; Writing – review & editing) E. J. A. Scherder (Conceptualization; Funding acquisition; Supervision; Writing – original draft; Writing – review & editing) H. C. Dijkerman (Conceptualization; Funding acquisition; Supervision; Writing – original draft; Writing – review & editing).

## Data Availability

Data sharing not applicable to this article as no data sets were generated or analysed during the current study.
